# Pinpointing the Band Gap Energy of GaAs by Fitting the Transmissive Part of the Absorption Spectrum with Bootstrap Error

**DOI:** 10.3390/ma18184266

**Published:** 2025-09-12

**Authors:** Mithun Bhowmick, Shuchismita Sarkar, Bruno Ullrich

**Affiliations:** 1 Department of Mathematical and Physical Sciences, Miami University, Middletown, OH 45042, USA; 2 Department of Applied Statistics and Operations Research, Bowling Green State University, Bowling Green, OH 43403, USA; ssarkar@bgsu.edu; 3 Ullrich Photonics LLC, Manistique, MI 49854, USA; bruno.ullrich@yahoo.com

**Keywords:** band gap, semiconductors, bootstrap resampling, gallium arsenide

## Abstract

Optical absorption in semiconductors is one of the most basic yet complex properties in crystalline semiconductors. There have been decades of studies on optical absorption with specific efforts towards understanding the accuracy of measurements. However, the interdependence of the parameters employed in the fit routines poses challenges in calculating consistent and reliable errors associated with the process when the band tailing states are involved, which is unavoidable in all practical measurements. This work examines room-temperature absorption measurements in undoped GaAs to report consistent error bars employing a bootstrap resampling analysis. The results show that (1) the transmissive part of the measured absorption contains all the information about the fit parameters for the entire spectrum and (2) it is possible to utilize statistical bootstrap sampling to determine error bars when interdependent fit parameters are present in the fit model.

## 1. Introduction

Optical absorption in crystalline and amorphous solids is critical for device applications [1]. In semiconductors, the mechanism refers to the absorption of a photon to excite an electron from the valence band to the conduction band, thereby creating an electron–hole pair. This fundamental process underpins applications in photovoltaics, photodetectors, and optoelectronics [2]. Below the band gap energy (*E*_g_), the optical absorption spectrum takes an exponential form, often noted as the Urbach tail, and provides insight into the electronic and structural disorder of materials [3,4]. The role of thermal effects in shaping the Urbach tail has been found to be insignificant [5].

The III–V compound semiconductor GaAs is known for its direct band gap and superior optoelectronic properties, making it a critical material in high-speed electronics, photovoltaic devices, and photonic applications. At room temperature, GaAs possesses a direct band gap of approximately 1.42 eV, meaning electrons can transition directly from the valence band to the conduction band without requiring phonon assistance [6]. This direct band gap results in a sharp absorption onset near *E*_g_ and a high absorption coefficient (>10^4^ cm^−1^), making GaAs highly efficient for absorbing photons in the visible and near-infrared range [6,7]. The absorption coefficient minimizes when the photonic energy (*E*) is lower than the *E*_g_ of a material. For these energies, the material shows easily measurable transmission, and therefore, this part of the spectrum could be called highly transmissive. The absorption coefficient increases rapidly with photon energy above the band gap due to strong interband transitions and reaches its maximum eventually. This part of the spectrum could be termed highly absorptive. The absorption edge of GaAs exhibits an Urbach tail with an Urbach energy typically on the order of a few meV for high-quality GaAs crystals [8,9]. Doping significantly alters the absorption characteristics of GaAs. For instance, heavily doped n-type GaAs reveals a blue shift of the absorption edge, known as a Burstein–Moss shift, due to the filling of low-energy conduction band states, reducing available final states for optical transitions [10]. Similarly, band tailing effects can become pronounced in disordered or nanostructured GaAs, such as in quantum wells or GaAs nanocrystals, which modify the effective band structure and absorption profile [9,11]. Advanced studies using techniques such as photoreflectance, photoluminescence excitation (PLE), and spectroscopic ellipsometry have further refined the understanding of GaAs optical absorption, enabling precise modeling of electronic transitions, effective mass parameters, and many-body effects [12,13].

Despite those vast research activities, a fundamental problem remains regarding the understanding of *E*_g_ in semiconductors such as GaAs at room temperature. When modeling optical measurements performed on materials with different degrees of purity, often, the fit routine returns optimized parameter values with associated uncertainties. These uncertainties are not always meaningful, nor could they be reported with confidence. Consequently, it becomes difficult to select the best fit among them or to state the “goodness of fit” with confidence. This is essentially a problem with variable degrees of doping that could be found in the diverse set of GaAs samples under investigation, where the shift of the absorption edge, the shape of the Urbach tail, and the maximum absorption can be expected to be different. A traditional fit routine aided with a statistical analysis thus seems practical to report error bars for the fit parameters.

In this study, we fit the optical absorption measurements of undoped GaAs with a statistical analysis on the fit parameters to attach errors associated with them. We also demonstrate that only the transmissive part of the optical absorption spectrum, essentially influenced by the tail states and governed by the exponential function, is required for reasonably accurate extraction of *E*_g_. The results give us error bars for each of the three fit parameters. The uniqueness of this work is in (1) the novelty of the fit routine used, where only the exponential part of the data was modeled to extract material parameters, and (2) the bootstrap resampling method used to statistically determine the errors associated with each of the three parameters.

## 2. Materials and Methods

The optical absorption spectra examined in this work were from Refs. [14,15,16]. These measurements are well separated in time, with the oldest one dating back to 1961 vs. the most recent reported in the year 2020 [14,15,16]. The reported optical absorption spectra were analyzed by fitting with Equations (1) and (2), as outlined below. This model, and the associated fit parameters were described with more details previously, and so only a brief overview will be included here [17,18]. The traditional fitting was performed using embedded user-defined functions in OriginPro 2023 (OriginLab Corporation, Northampton, MA, USA). To fit an optical absorption in direct band gap semiconductors, a modified Urbach rule has been found to accurately describe the affair [18]. In that model, the highly transmissive part, where the absorption coefficient rapidly drops, i.e., for *E* < *E*_g_ the spectrum is described as follows:

(1) $$\alpha \left(E\right)\;=\;A\sqrt{\frac{kT}{2\sigma }}e^{\frac{\sigma [E\;-\;E_{g}]}{kT}}$$

where $\alpha \left(E\right)$ is optical absorption coefficient vs. *E*, A is a parameter related to the saturation of absorption, *k* is Boltzmann constant, *T* is the temperature, and *E*_g_ and σ are fit parameters representing the band gap energy and the slope coefficient of the Urbach decay, respectively. When the photonic energy is equal or greater than the *E*_g_ of the material, the absorption is modeled by the density of states function. Thus, for *E* ≥ *E*_g_, the expression could be written as follows:

(2) $$\alpha \left(E\right)\;=\;A\sqrt{E-E_{g}+\frac{kT}{2\sigma }}$$

Note that the fit parameter *A* is not merely a phenomenological entity but can be interpreted as a measure of the saturation limit of $\alpha \left(E\right)$, expressed by the following [14,15,17]:

(3) $$A\;=\;2\alpha_{f}\frac{{\left(2m_{r\;}^{*}\right)}^{3/2}}{nm_{e}^{*}h}$$

where $\alpha_{f}$ is the fine structure constant and $m_{r}^{*}$ is the reduced effective mass, defined as follows:

(4) $$m_{r}^{*}\;=\;\frac{m_{e}^{*}m_{h}^{*}}{m_{e}^{*}\;+\;m_{h}^{*}}$$

where $m_{e}^{*}$, $m_{h}^{*}$ are the electron and hole effective masses, respectively; *n* is the refractive index in the vicinity of *E_g_*; and *h* is the Planck constant. With $m_{e}^{*}/m_{0}$ = 0.068 and $m_{h}^{*}/m_{0}$ = 0.5, where $m_{0}$ is the free electron mass, we previously found that *A* = 1.5 × 10^4^ eV^−1/2^ cm^−1^ [17,18].

The three fit parameters, *A*, *σ*, and *E*_g_, were examined in this work, with a focus on precisely extracting *E*_g_ from the fit results with error bars. OriginPro 2023 fits were generated via user-defined nonlinear curve fitting routines based on Levenberg–Marquardt algorithm, which is a standard approach in software applications for solving curve fitting problems [19]. For the bootstrap analysis, R software (R-4.4.2) was used. During the optimization of the OriginPro 2023 fits, it was found that due to interdependence of the parameters, more than one “best fit” exist. To obtain robust confidence intervals for the fit parameters, bootstrap resampling was employed to the population described in the data. Bootstrap sampling is a powerful resampling technique used to estimate the sampling distribution of a statistic by repeatedly drawing samples, with replacement, from the observed data [20]. Each bootstrap sample is of the same size as the original dataset and may include repeated observations. By calculating the statistics of interest (e.g., the mean, median, or regression coefficient) for each bootstrap sample, we obtain a distribution of the statistic across many resamples. This empirical distribution can then be used to construct confidence intervals, typically by identifying percentiles from the bootstrap distribution (e.g., the 2.5th and 97.5th percentiles for a 95% confidence interval). Bootstrap methods are particularly useful when theoretical confidence intervals are difficult to derive or when the underlying distribution of the data is unknown.

In this study, the resulting bootstrap distributions were often found to be skewed and multimodal. Under such circumstances, confidence intervals based on the simple percentile method may be misleading. To address this, we employed the bias-corrected and accelerated (BCa) bootstrap confidence interval method, which adjusts for both bias and skewness in the bootstrap distribution [21]. The method incorporates two correction terms: a bias-correction factor, which shifts the interval to account for the difference between the bootstrap mean and the observed statistic, and an acceleration factor, which adjusts for skewness. By recalibrating the percentile cutoffs using these adjustments, BCa intervals generally provide more accurate coverage probabilities than symmetric percentile intervals, particularly for nonlinear estimators or when sample sizes are limited. To ensure stability of the uncertainty estimates, we repeated the bootstrap procedure with different numbers of resamples (n_boot_ = 1000, 2000, and 3000). The resulting confidence intervals were consistent across these sample sizes, indicating that the bootstrap distributions had converged and that further increases in n_boot_ would not materially affect the reported intervals.

Based on the results from the first set of bootstrap sampling, a second approach examined the viability of reducing the number of fit parameters from 3 to 2 by treating *A* as a constant fixed to its theoretically calculated value. For this part, bootstrap analysis was run only for the highly transmissive part of the spectrum (when *E* < *E*_g_) with a fixed *A* and variable *σ*, *E*_g_.

## 3. Results and Discussion

**Figure 1:**
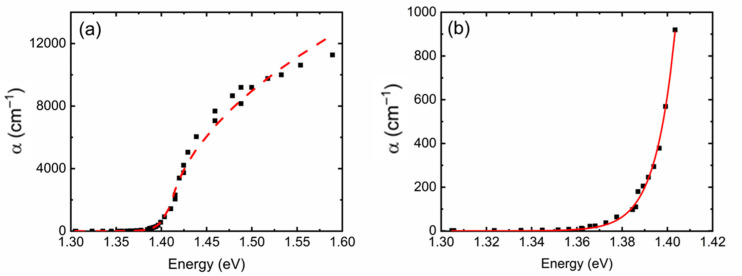
Data (symbols) and fits (lines) for (**a**) the entire absorption spectrum and (**b**) only the transmissive part from Ref. [14].

Example OriginPro 2023 fits, resulting from Ref. [14], are presented in Figure 1, where the two regions of the optical absorption spectrum have been modeled. Employing the model for the whole spectrum (Figure 1a) and for the transmissive part (Figure 1b) generated fit parameters presented in Table 1 below. The fit parameters are reasonably close with small uncertainty values, which were extracted from the algorithm directly.

While in most cases the fits represent an accurate picture of the associated errors in the fitting routine, in certain cases, they do not actually return any meaningful values. For example, the error in *σ* for the entire spectrum notes 8.76 × 10^−17^, which just means that the fit returned a tiny uncertainty but does not provide any quantitative way to determine the confidence in that error. Nevertheless, the fit parameters listed in Table 1 confirm that the transmissive part of the spectrum indeed contains all the information necessary to extract the band gap of GaAs. It is therefore important to examine the accuracy of the three fit parameters, *A*, *σ*, and *E*_g_.

It is expected to have a wider range of *A* values for a variety of GaAs samples. While *E*_g_ is one of the most discussed parameters in direct band gap semiconductors, the physical interpretation of *A* has not been commented on until recently, where for the first time, it was analytically linked to the absorption limit via effective electron density of states [17]. The values of *A* for several III–V materials have recently been calculated employing Equation (3), where it has also been noted that the measurement quality directly impacts its value [17]. Apart from poor measurements, there could be other factors affecting an optimal fit, such as poor initial parameter values, difficulty in locating an absolute minimum in χ^2^ calculation, and cases where multiple values of the same parameter can provide comparable solutions. For example, a range of values could be attributed to *A* for identical fits while attempting to model a certain measurement using Equations (1) and (2) through a standardized fit routine such as OriginPro 2023. The parameter *σ* has been linked to the steepness of the absorption edge and is also directly impacted by structural disorder, doping, or the phonon dynamics [17]. Considering that real materials always contain some disorders, it is understandable that by modeling absorption spectra, the optimization of *A*, *σ*, and *E*_g_ is required, while some interdependence of these parameters ought to be considered [17].

**Figure 2:**
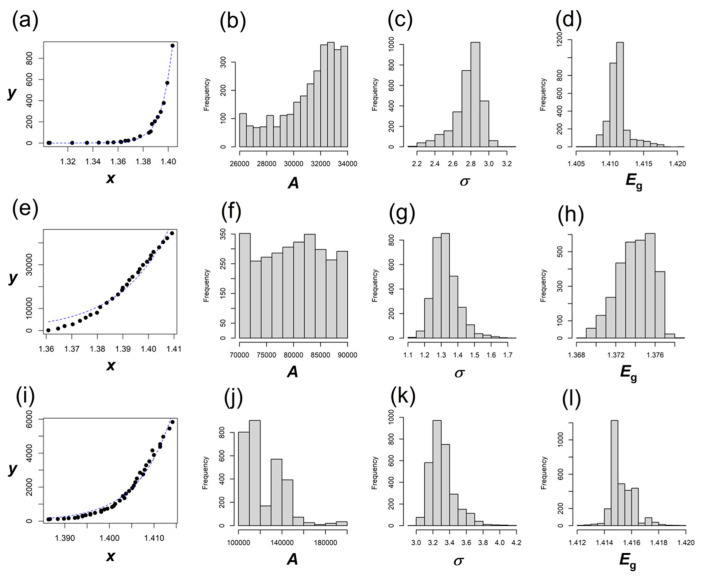
Data (symbols) with fits (lines) for the transmissive part (**a**,**e**,**i**), and corresponding bootstrap distributions of *A*, *σ*, and *E*_g_ for measurements from Ref. [14] (**b**–**d**), Ref. [18] (**f**–**h**), and Ref. [19] (**j**–**l**) when *E* ≥ *E*_g_. In this figure *x*, *E* and *E*_g_ are in eV, whereas *y* and *A* are in eV^−1/2^ cm^−1^.

**Figure 3:**
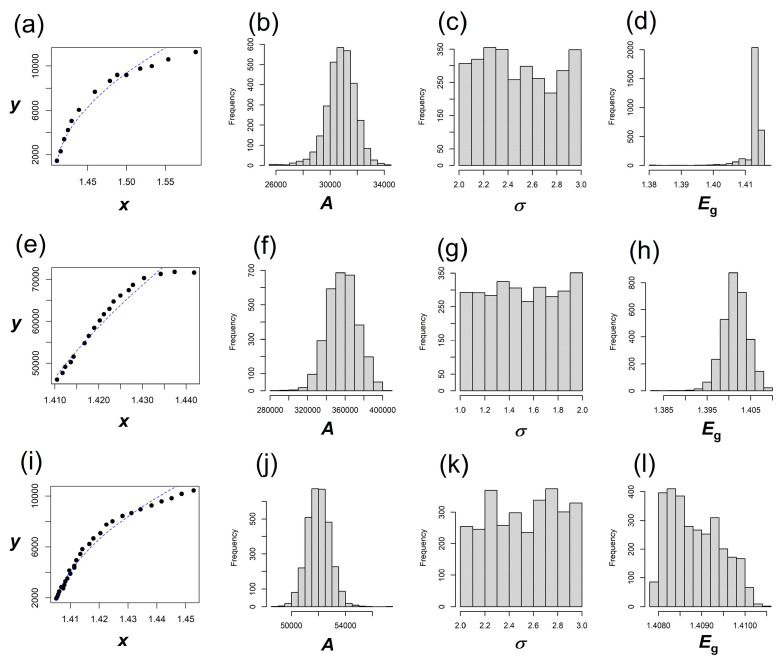
Data (symbols) with fits (lines) for the absorptive part (**a**,**e**,**i**), and corresponding bootstrap distributions of *A*, *σ*, and *E*_g_ for measurements from Ref. [14] (**b**–**d**), Ref. [18] (**f**–**h**), and Ref. [19] (**j**–**l**) when *E* ≥ *E*_g_. In this figure *x*, *E* and *E*_g_ are in eV, whereas *y* and *A* are in eV^−1/2^ cm^−1^.

**Figure 4:**
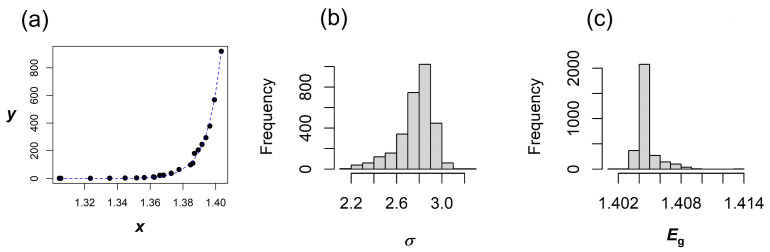
Data (symbols) with fit (line) when bivariate bootstrap distribution is determined for fixed A = 15,000 (**a**), bivariate bootstrap distributions of *σ* (**b**) and *E*_g_ (**c**) for the transmissive part of the GaAs absorption from Ref. [14]. In this figure *x* and *E*_g_ are in eV, whereas *y* and *A* are in eV^−1/2^ cm^−1^.

For each dataset analyzed in this study, two separate bootstrap analyses were performed. The first focused on optimizing the fit for the transmissive part of the spectrum (*E* < *E*_g_), while the second examined the remaining part of the spectrum (*E* ≥ *E*_g_). To assess stability, bootstrap resampling was carried out with n_boot_ = 1000, 2000, 3000, and the resulting confidence intervals demonstrated consistency across these sample sizes. The relative change in the estimated lower and upper bounds between n_boot_ = 2000 and n_boot_ = 3000 is less than 0.005 for parameters *A* and *E*_g_. For parameter *σ*, the corresponding upper bound is 0.01 (in case of *E* ≥ *E*_g_, Ref. [14], where the *σ* exhibits little influence on the fit of the equation). The associated bootstrap distributions are shown in Figure 2, Figure 3 and Figure 4. The point estimates of the parameters, along with their bootstrap confidence intervals, are summarized in Table 2 (for *E* < *E*_g_) and Table 3 (for *E* ≥ *E*_g_). In these tables, the n_boot_ column indicates the bootstrap sample size. The parameter estimates obtained from the original data are denoted as PE_original_. The bias-corrected and accelerated (BCa) bootstrap confidence intervals are reported as (LB_BCa_, UB_BCa_). Ninety-five percent BCa confidence intervals are provided for all three parameters, *A*, *σ*, and *E*_g_, under each value of n_boot_.

For example, for the bootstrap sample corresponding to Ref. [14], a nonlinear least square optimization was applied on *σ* and *E*_g_ after varying *A* over a grid of 26,000 to 34,000. The results are presented in Figure 2a–d, with estimated parameters listed in rows 1–7 of Table 2. The value of *A* from this run is found to be 32,380, with the LB_BCa_ and UB_BCa_ to be 28,299 and 34,000, respectively (n_boot_ = 3000). The second bootstrap examined the remaining part of the spectrum (when *E* ≥ *E*_g_), corresponding to the same data [14], and is presented in Figure 3a–d, with the estimated parameters listed in rows 1–7 of Table 3. The estimated *A* from this part is 30,927, with the LB_BCa_ and UB_BCa_ as 28,632 and 32,791, respectively (n_boot_ = 3000). The estimated *σ* and *E_g_*, when *E* < *E*_g_, are 2.83 and 1.411, respectively, and they showed a much narrower confidence interval, as could be seen in Table 2 (rows 1–7). These values are not very different from the results for *E* ≥ *E*_g_ (*σ* = 2.608, *Eg* = 1.413 eV). Likewise, absorption data from Ref. [15] was also examined through separate sets of bootstrap sampling for *E* ≥ *E*_g_ and *E* ≥ *E*_g_ parts of the spectra, and corresponding results could be found in Figure 2 and Figure 3e–h, with estimated parameters listed in Table 2 and Table 3 (rows 8–14). Finally, for Ref. [16], the bootstrap results are presented in Figure 2 and Figure 3i–l, with the parameter values listed in Table 2 and Table 3 (rows 15–21). The band gaps extracted from the three datasets for the transmissive part (when *E* < *E*_g_) are very close to those extracted for *E* ≥ *E*_g_. For Refs. [14,15,16], the comparisons of band gaps could be noted as follows: 1.411 eV vs. 1.413 eV, 1.375 eV vs. 1.402 eV, and 1.415 eV vs. 1.410 eV, respectively.

Last but not the least, a bootstrap analysis examined the model with a fixed *A* at its theoretical value of 15,000 and tested the confidence levels for *σ* and *E*_g_. The results, presented in Figure 4, show unimodal distribution for *σ* and *E*_g_ with 95% BCa confidence intervals in the range 2.47 to 3.04, and 1.402 to 1.406 eV, respectively, narrowing the parameters further down. The accuracy of the extracted *E*_g_ values could be discussed considering reported measured/calculated *E*_g_ from the literature. In undoped GaAs, some of the values reported in the literature are 1.424 eV [6], 1.4179 eV [16], 1.4287 eV ± 0.008 eV [22], 1.424 eV ± 0.002 eV [23], and 1.422 eV [24]. These values agree well with the *E*_g_ extracted from the transmissive part of the spectrum by bootstrap sampling results reported in Table 1, Table 2 and Table 3.

To summarize the results, the general applicability of the bootstrap analysis was confirmed with a total of three datasets from the literature [14,15,16]. While *σ* and *E*_g_ values stayed within narrower ranges when compared between transmissive vs. absorptive parts of the spectrum, the same for *A* was broader. One potential solution to this issue could be to fix *A* to its theoretical value. The *E*_g_ values extracted from the transmissive part of the spectrum were very close to the previously reported values from the literature. The bootstrap error analysis noted two important features in the fit routines. For the transmissive part, the fit easily pinpoints *σ* and *E*_g_ but struggles to optimize *A* to a narrow range of values. This could be interpreted as existence of multiple fits of similar accuracy for each of those values returned for *A*. On the other hand, when *E* ≥ *E*_g_, the model converges well for *A* and *E*_g_, but *σ* remains unoptimized, leading to multiple similarly efficient fits for each of the values of *σ*.

Physically, the challenges faced by the analytical scheme to converge on a narrow range for *A* could be explained by the theoretical definition of the same. *A* has been defined as the absorption saturation parameter linked to the effective electron density of states. For *E* ≥ *E*_g_, the absorption increases to the maximum before saturating. When fitting this part of the spectrum, *A* becomes relevant and “visible” to the fit routines and bootstrap error algorithm, thereby comfortably returning the parameter values with uncertainty. The fit parameter *σ* defines the steepness of the Urbach tail states of the absorption spectrum and therefore is a measure of purity and disorder of the material. It is thus understandable to have a clearer range and convergence for *σ* when *E* < *E*_g_, where the slope of the absorption curve becomes more important. This part of the spectrum is practically transparent with little absorption, which could be the reason for the challenges found in the convergence of *A* for this part of the fit. The fact that *σ* and *A* are both connected to material properties leads to the interdependence of those parameters.

While working with traditional fit routines such as OriginPro 2023, the deviation from the theoretical value of *A* with the one found from the fit could be explained by the fact that real materials are affected by defects, changing the absorption spectrum. Due to these factors and since typical UV-VIS spectrophotometers lack resolution to provide enough data points in the transmissive part of the spectrum, values within a factor of 2 were common and have been considered adequate when discussing *A* [17]. The values of A reported in this work certainly meet that criterion. Moreover, it has been shown in GaAs and GaSb that a measured transmission spectrum with more data points in the *E* ≥ *E*_g_ region results in values of *A* that are closer to the theoretical predictions [17]. Nevertheless, the fit routine using OriginPro and the bootstrap sampling have successfully pinpointed the *E*_g_ of the material, which was one of the primary goals in this work. The bootstrap sampling not only established the error bars in all the three fit parameters but also confirmed the consistency of confidence levels. It is captivating to note that by fitting only a small part of the spectrum, one can extract these parameters, particularly *E*_g_. It will be interesting to apply this analysis technique to multiple groups of direct band gap semiconductors, eventually leading to the development of a high-throughput characterization tool.

## 4. Conclusions

In summary, by theoretically fitting only the transmissive part of the optical absorption spectrum, it was possible to extract the *E*_g_ of GaAs accurately. The other two fit parameters (*σ*, *A*), linked to the level of disorder in the material, could also be found with reasonable accuracy. Bootstrap resampling successfully attached error bars to the fit parameters and confirmed the consistency of the confidence levels.

## Figures and Tables

**Table 1:** Fit parameters corresponding to the whole spectrum (first row) and only the low energy, highly transparent part of the spectrum (second row) corresponding to Figure 1.

α (*E*) (cm^−1^)	*A* (eV^−1/2^ cm^−1^)	*σ*	*E*_g_ (eV)
*E* < *E*_g_ and *E* ≥ *E*_g_	29,401 ± 705	2.408 ± 8.76 × 10^−17^	1.412 ± 1.43 × 10^−3^
*E* < *E*_g_	28,944 ± 315	2.833 ± 5.9 × 10^−2^	1.410 ± 4.7 × 10^−4^

**Table 2:** Results for the bootstrap sampling when *E* < *E*_g_. Values for *A* and *E*_g_ are expressed in eV^−1/2^ cm^−1^ and eV, respectively, while *σ* is dimensionless.

		Ref. [14]			Ref. [15]			Ref. [16]		
n_boot_		*A*	*σ*	*E*_g_	*A*	*σ*	*E*_g_	*A*	*σ*	*E*_g_
	PE_original_	32,380	2.83	1.411	83,534	1.30	1.375	112,813	3.27	1.415
1000	LB_BCa_	28,045	2.55	1.408	73,828	1.14	1.372	100,023	3.03	1.413
	UB_BCa_	34,000	3.09	1.415	90,000	1.43	1.377	141,441	3.51	1.416
2000	LB_BCa_	28,183	2.56	1.408	74,064	1.15	1.372	100,070	3.02	1.412
	UB_BCa_	34,000	3.09	1.415	90,000	1.43	1.377	141,942	3.51	1.416
3000	LB_BCa_	28,299	2.58	1.408	74,064	1.15	1.372	100,000	3.02	1.412
	UB_BCa_	34,000	3.09	1.414	90,000	1.43	1.377	142,442	3.54	1.416

**Table 3:** Results for the bootstrap sampling when *E* ≥ *E_g_*. Values for *A* and *E_g_* are expressed in eV^−1/2^ cm^−1^ and eV, respectively, while *σ* is dimensionless.

		Ref. [14]			Ref. [15]			Ref. [16]		
n_boot_		*A*	*σ*	*E*_g_	*A*	*σ*	*E*_g_	*A*	*σ*	*E*_g_
	PE_original_	30,927	2.61	1.413	356,704	1.40	1.402	51,943	2.91	1.408
1000	LB_BCa_	32,001	2.48	1.380	320,962	1.01	1.397	50,074	2.73	1.408
	UB_BCa_	33,975	3.00	1.413	382,611	1.93	1.407	53,459	3.00	1.408
2000	LB_BCa_	28,519	2.10	1.391	319,407	1.01	1.396	50,158	2.74	1.408
	UB_BCa_	32,737	3.00	1.414	383,387	1.93	1.407	53,509	3.00	1.408
3000	LB_BCa_	28,632	2.12	1.386	318,836	1.01	1.396	50,210	2.74	1.408
	UB_BCa_	32,791	3.00	1.414	382,582	1.93	1.407	53,527	3.00	1.408

## Data Availability

The original contributions presented in this study are included in the article. Further inquiries can be directed to the corresponding author.
